# A Novel *PPP1R12A* Splice‐Site Variant Identified in a Female Fetus With Perineal Hamartoma

**DOI:** 10.1002/pd.70183

**Published:** 2026-05-21

**Authors:** Shiyu Chen, Xi Tan

**Affiliations:** ^1^ Department of Ultrasonic Medicine Sichuan University West China Second University Hospital Chengdu Sichuan China; ^2^ Sichuan University Key Laboratory of Obstetric and Gynecologic and Pediatric Diseases and Birth Defects Chengdu Sichuan China; ^3^ Department of Obstetrics and Gynecology Sichuan University West China Second University Hospital Chengdu Sichuan China

## Abstract

- What is already known about this topic?
  ◦ *P0050P1R12A*‐related UBMS is characterized by midline brain anomalies and urogenital defects; and reported urogenital anomalies predominantly affect male (46, XY) individuals, while prenatal findings in female (46, XX) individuals remain poorly defined.
- What does this study add?
  ◦ We report a de novo splice‐site *PPP1R12A* variant in a female fetus with perineal hamartoma. This observation may represent a novel prenatal manifestation in female individuals.

## 1 Fetal Phenotype

**FIGURE 1:**
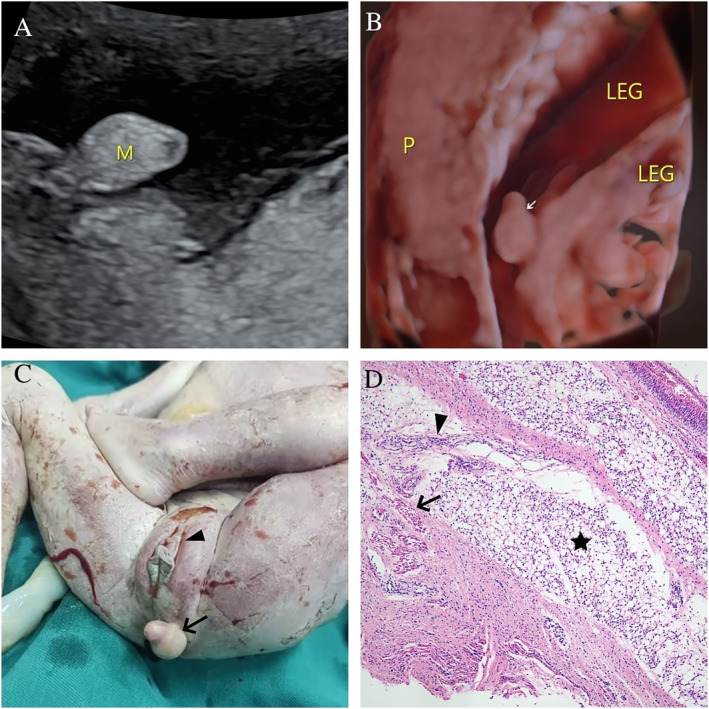
(A, B) Prenatal two‐ and three‐dimensional ultrasound images of a perineal mass at 29 weeks; (C) post‐delivery image showing a pedunculated perineal mass (arrow) located inferior and to the left of the normal female external genitalia (arrowhead); (D) histology showing disorganized skeletal muscle (arrow), nerve bundles (arrowhead), and adipose tissue (asterisk), beneath the skin…, supporting the diagnosis of hamartoma. M, mass; P, placenta.

A 33‐year‐old woman (gravida 2, para 1) underwent regular prenatal care. At 24 weeks of gestation, ultrasonography revealed a 14 × 8 mm solid perineal mass in the fetal perineal region, which increased to 17 × 11 mm at 29 weeks (Figure 1A, B). No additional structural anomalies were identified, with normal intracranial structures and urinary system. Given the perineal location, differential diagnoses included abnormalities of external genital development and sacrococcygeal teratoma. Fetal MRI confirmed the ultrasound findings, demonstrating normal female external genitalia and an isolated perineal mass of uncertain etiology. The parents reported an unremarkable family history and had previously delivered a healthy daughter. After counseling, pregnancy was terminated at 33 weeks (Table 1A).

**TABLE 1A:** Clinical data.

Case	Parental details			Gestation at diagnosis	Phenotypes (HPO terms)	Obstetric history	Family history	Outcome
1	Maternal	Age	33	24 weeks	Hamartoma (HP: 0010566)	G2P1	Unremarkable	Induced labor; perineal mass confirmed; no additional external anomalies; prenatal imaging showed no additional intracranial or urinary tract anomalies
		Ethnicity	Asian					
	Paternal	Age	38					
		Ethnicity	Asian					

The delivered fetus exhibited a phenotypically female appearance consistent with the karyotype (46, XX), and showed normal female external genitalia and a clearly visible anus. A 20 × 15 mm pedunculated lesion beneath the left labium majus was confirmed as a hamartoma on histology (Figure 1C, D); no autopsy was performed.

## 2 Diagnostic Method

We conducted trio‐based exome sequencing (trio‐ES) on fetal amniotic fluid and parental peripheral blood DNA using the Illumina NovaSeq 6000 platform with 150‐bp paired‐end reads. Sequencing reads were aligned to the human reference genome GRCh38/hg38 using the Burrows‐Wheeler Aligner (BWA, v0.7.17), and variant calling and annotation were conducted by applying the ENLIVEN variant interpretation system (Berry Genomics). We confirmed the variants identified through trio‐ES by Sanger sequencing.

## 3 Diagnostic Results and Interpretation

Karyotype analysis demonstrated a normal female karyotype (46, XX), and chromosomal microarray analysis (CMA) revealed no clinically significant copy number variants. Trio‐ES identified a heterozygous de novo splice‐site variant in *PPP1R12A* (NM_001244990.2:c.1239+2T>G), which was confirmed by parental testing (Table 1B). The variant was absent from population databases, including 1000 Genomes, ExAC, and gnomAD. Nomenclature was validated using VariantValidator (see Supporting Information S1) [1]. SpliceAI predicted a high probability of donor loss (DS_DL = 0.98) for the NM_001244990.2:c.1239+2T>G variant, affecting the canonical donor + 2 splice site. In silico analysis supported disruption of normal splicing, consistent with loss of function as the established disease mechanism for *PPP1R12A*‐related diseases. In the absence of RNA‐based functional validation, the transcript consequence remains unconfirmed. Therefore, PVS1 was applied at a strong level (PVS1_Strong), in line with ClinGen recommendations for predicted splice variants [2, 3]. No other potentially pathogenic variants were identified. According to the ACMG/AMP 2015 guidelines, and based on the combined evidence (PVS1_Strong, PS2_Moderate, PM2_Supporting), the variant was classified as likely pathogenic. To our knowledge, the *PPP1R12A* c.1239+2T>G variant has not been previously reported in a female fetus with perineal hamartoma.

**TABLE 1B:** Genetic findings.

Procedure (gest age)	Direct/culture?	Performed test	Secondary confirmatory test	Gene (name; REFSEQ)	Known disease (OMIM)	Variant	ACMG classification	Criteria applied	Inheritance & zygosity	Interpretation
Amniocentesis (24^+ 5^ weeks)	Direct	Trio‐based exome sequencing	Sanger sequencing	*PPP1R12A* (NM_001244990.2)	UBMS (OMIM: 618820)	NM_001244990.2:c.1239+2T>G	Likely pathogenic	PVS1_Strong, PS2_Moderate, PM2_Supporting	Heterozygous, De novo	Likely pathogenic; predicted splice‐altering variant; no RNA validation

## 4 Discussion

*PPP1R12A* encodes myosin phosphate target subunit 1 (MYPT1), a key regulatory component of the myosin phosphatase complex that modulates actomyosin contractility [4, 5]. *PPP1R12A* expression has been localized to the prosencephalic neural folds and in the developing lower urinary tract—including the genital tubercle, urethra, and bladder—during critical periods of brain and urogenital morphogenesis [6].

Pathogenic loss‐of‐function variants in *PPP1R12A* have been associated with urogenital and/or brain malformation syndrome (UBMS), characterized primarily by abnormalities of the urogenital system and midline brain malformations [6, 7]. In the series reported by Hughes et al., urogenital anomalies were identified in 9 of 12 cases and brain malformations in 5 of 12. The urogenital phenotype was particularly prominent in reported male (46, XY) individuals, with findings ranging from undervirilization, micropenis, and hypospadias to bifid scrotum, female‐appearing external genitalia, and gonadal dysgenesis. Brain abnormalities were observed in a smaller subset of cases, often involving the holoprosencephaly spectrum or other midline brain anomalies. In contrast, the phenotype in female (46, XX) individuals remains less well defined, as female cases are few and genitourinary assessment has often been limited [6]. In addition to the previously described UBMS phenotype, recent reports suggest possible broader developmental involvement in *PPP1R12A*‐related disorders, particularly involving caudal developmental structures, including anorectal malformations and related anomalies [8, 9]. Congenital perineal masses, including hamartomas, are frequently associated with urogenital, anorectal, or external genital anomalies; in the review by Zhang et al., such abnormalities were present in 14 of 23 cases (60.9%) [10]. This is consistent with embryology, as the urorectal septum partitions the cloaca into the primitive urogenital sinus and rectum during weeks 6–7 of development, while the perineum arises from its superficial portion [11]. From this perspective, the present fetus may represent a perineal developmental anomaly that is potentially related to the broader spectrum of caudal developmental involvement in *PPP1R12A*‐related disease. Although prenatal ultrasound showed no intracranial abnormalities and the external genitalia and anus appeared normal after delivery, the lack of autopsy precludes the exclusion of additional *PPP1R12A*‐related brain, genitourinary, anorectal, or other internal anomalies. Phenotypic characterization in this case was therefore incomplete. Thus, this case may point to broader developmental involvement in *PPP1R12A*‐related disease; however, additional prenatally ascertained cases are needed before perineal hamartoma can be considered an established feature of the phenotype.

In conclusion, we report a female fetus with perineal hamartoma carrying a de novo heterozygous *PPP1R12A* variant (c.1239+2T>G). This case raises the possibility that perineal hamartoma may be relevant to the prenatal phenotype of *PPP1R12A*‐related disease, although the current evidence remains insufficient to consider it an established feature. Our findings also underscore the value of trio‐ES in the evaluation of unexplained fetal perineal abnormalities.

## Funding

The authors have nothing to report.

## Ethics Statement

The authors have nothing to report.

## Consent

Written parental consent was obtained to share the details and images of this Case Report.

## Conflicts of Interest

The authors declare no conflicts of interest.

## Supporting information


Supporting Information S1

## Data Availability

The data that support the findings of this study are available on request from the corresponding author. The data are not publicly available due to privacy or ethical restrictions.
