# Supplementary material for: A Novel PPP1R12A Splice‐Site Variant Identified in a Female Fetus With Perineal Hamartoma
Source: Prenat Diagn. 2026 May 21;46(8):1290–3. doi: 10.1002/pd.70183 (PMC13377262; doi:10.1002/pd.70183)
Supplement: Supplementary file 1 — Supporting Information S1 [file PD-46-1290-s001.pdf]

VariantValidator

Submitted Variant

NM_001244990.2:c.1239+2T>G

- Selected genome build: GRCh38
- Map location: 12q21.2-q21.31
- CCDS ID: CCDS58260.1

Versions

- VariantValidator 3.0.2.dev178+g91cc31edf
- vv_hgvs 2.2.1.dev22+gebef587af
- VVDb vvdb_2025_3
- VVTA vvta_2025_02
- VVSeqRepo VV_SR_2025_02/master

Recommended Variant Descriptions

1. HGVS guidelines recommend using genomic and transcript descriptions in all publications
2. Use of the three- or one-letter amino acid alphabet is optional, but three-letter is recommended

Genomic descriptions

Reference Sequence Type	Variant Description
Chromosomal GRCh37	NC_000012.11:g.80211172A>C
Chromosomal GRCh38	NC_000012.12:g.79817392A>C

Transcript and protein descriptions

Reference Sequence Type	Variant Description
Transcript	NC_000012.12(NM_001244990.2):c.1239+2T>G
Protein three letter code	NP_001231919.1:p.?
Protein single letter code	NP_001231919.1:p.?

Gene Information

Attribute	Identifier	Source
Symbol	PPP1R12A	HGNC
Name	protein phosphatase 1 regulatory subunit 12A	HGNC
HGNC ID	HGNC:7618	HGNC

MANCHESTER 1824
The University of Manchester

Copyright © 2016-2026 VariantValidator Contributors

UNIVERSITY OF LEICESTER
